# Oxytocin Pathway Gene (*CD38, OXTR*) Variants Are Not Related to Psychosocial Characteristics Defined by Strengths and Difficulties Questionnaire in Adolescents: A Field School-Based Study

**DOI:** 10.3389/fpsyt.2021.714093

**Published:** 2021-08-09

**Authors:** Sergey Tereshchenko, Edward Kasparov, Svetlana Zobova, Marina Smolnikova, Lidia Evert, Nadezhda Semenova, Olga Zaitseva, Margarita Shubina, Nina Gorbacheva, Ludmila Lapteva

**Affiliations:** ^1^Federal Research Center “Krasnoyarsk Science Center of the Siberian Branch of the Russian Academy of Sciences”, Research Institute of Medical Problems of the North, Krasnoyarsk, Russia; ^2^Krasnoyarsk State Medical University, Krasnoyarsk, Russia

**Keywords:** oxytocin, CD38, OXTR, rs3796863, rs53576, gene polymorphism, adolescents, psychosocial characteristics

## Abstract

**Background:** CD38 is a transmembrane glycoprotein that regulates oxytocin (OT) production and influences social interactions. The oxytocin receptor (OXTR) has been studied intensively regarding its association with human psychosocial functions. Many studies have demonstrated a link between *CD38* rs3796863 and *OXTR* rs53576 polymorphic regions and psychosocial characteristics as well as various psychiatric disorders in adolescents. Some studies, however, have reported null findings.

**Methods:** The Strengths and Difficulties Questionnaire (SDQ) is a brief psychopathologic screening tool recommended for detecting psychosocial problems and psychiatric disorders in adolescents. The current field school-based study, conducted among urban Siberian adolescents (*n* = 298 aged 12–18), explored the SDQ scales in relation to polymorphisms of the *CD38* and the *OXTR* genes (rs3796863 and rs53576, respectively).

**Results:** None of the studied genotypes were associated with the SDQ results for the complete sample with presumed statistical power as 0.80 to detect a medium-size effect (Cramer's V = 0.3) at α = 0.0083. *Post-hoc* analysis in subgroups showed that OT pathway high activity may cause some negative consequences, such as emotional instability in older (aged 15–18) adolescent boys who are carriers of the rs53576 GG variant.

**Conclusion:** Variations at the CD38 rs3796863 and OXTR rs53576 loci were not associated with psychosocial characteristics of adolescents assessed with the SDQ. In studies with a similar design, we recommend replication with larger samples and greater power to detect small effects, especially in age–sex subgroups of adolescents.

## Introduction

Oxytocin (OT) is a nonapeptide neurohormone mainly produced in the supraoptic and paraventricular nuclei of the hypothalamus. Large-cell oxytocin-producing neurons of the hypothalamus have axonal connections with the posterior lobe of the pituitary gland, where OT is deposited and subsequently released into the bloodstream with the implementation of its peripheral action occurring *via* the activation of specific receptors. Additionally, OT has a direct central effect on various parts of the brain, mediated through its dendritic release followed by diffusion into adjacent areas.

The primary hormonal role of OT is to regulate the processes of gestation, labor, and lactation, as well as the establishment of social bonds from infancy through to adolescence and adult life. Its central action constitutes an essential part of cognitive, emotional, and behavioral processes (1). Moreover, OT takes part in the regulation of eating and sexual behavior (2, 3), mechanisms of visceral hypersensitivity (4), and pain perception (5).

In recent years, the genetic aspects of the regulation of the production and reception of OT in various psychopathological conditions in adolescents have attracted the close attention of researchers. Studies of the genetic basis for the oxytocinergic system have mainly focused on single nucleotide variants of the *OXTR* gene (rs53576, rs2254298), the *OXT* gene (rs2740210, rs4813627, rs4813625), and the *CD38* gene (rs3796863, rs6449197) (6). Many studies have shown the association between these variants and aggressiveness, poor tolerance to psychological stress (7), as well as suicidal tendencies (8), problems with behavior, the parent–child relationship (9, 10), and attention deficit hyperactivity disorder (11). A large number of studies have demonstrated the existence of a link between different polymorphic regions of the *OXTR* as well as the *CD38* genes and various psychiatric diseases, including autism spectrum disorders [analyzed in detail in the reviews by Feldman et al. (6) and Cataldo et al. (12)].

CD38 is a transmembrane glycoprotein with adenosine diphosphateribosyl-ribosyl cyclase activity, which plays a vital role in regulating hormonal production and cell differentiation, and migration (13). CD38 is expressed in hematopoietic cells (B- and T-lymphocytes) and hypothalamic neurons. The first reports on the ability of CD38 to regulate OT production through Ca^2+^-signaling and influence social interactions were published by Jin et al. in 2007 (14). Subsequent studies have shown that *CD38* knockout mice have an outstanding reduction in OT production (15). The single nucleotide polymorphism rs3796863 (A > C) located in intron 7 of the *CD38* gene, which is has been mapped in the 4p15 chromosomal region (16). It is assumed that the rs3796863 A allele variant is associated with high expression of CD38, increased plasma oxytocin concentration, and a pronounced level of social sensitivity (17).

The oxytocin receptor (OXTR) belongs to class I of the G-protein family, has seven transmembrane domains, and is encoded by the *OXTR* gene located in the 3p25–3p26.2 chromosomal region. The *OXTR* gene contains three introns and four exons; the role of the rs53576 (G > A) polymorphic variant, localized in the third exon, has been studied intensively regarding its association with human social functions (6). It is not entirely clear how the *OXTR* rs53576 variant is translated into phenotypic variations. It has been assumed that the rs53576 variant influences the methylation of the *OXTR* gene: the carriage of the G allele may be associated with a low level of methylation and subsequent high transcription of the gene, enhanced expression of the OT receptor, and an increased level of social sensitivity (18–21).

The Strengths and Difficulties Questionnaire (SDQ) was developed by Goodman et al. (22) as a brief psychopathological screening tool and is recommended to detect and classify psychosocial problems in adolescents. The SDQ is now very widely used in clinical practice and scientific research because of its brevity, reliability, and ability to assess various aspects of the psychosocial state in adolescents. The validity of the SDQ in clinical setting was confirmed by numerous studies (23–25). An undoubted advantage of the SDQ is also its wide availability: currently, it has been translated into more than 80 languages. It is also freely available on the developers' website (https://sdqinfo.org/), making cross-cultural comparisons possible. The Russian version of the SDQ has been thoroughly validated by Ruchkin et al. (26) and Slobodskaya et al. (27) on a sample group of Siberian schoolchildren (Novosibirsk, Russia).

To the best of our knowledge, there are no field studies on the effect of OT gene polymorphism on the psychosocial characteristics of adolescents in an unbiased school sample using the SDQ questionnaire. In this exploratory study, we adopted a genetic approach to analyze psychosocial characteristics in a sample of adolescents, focusing on two polymorphisms of the *CD38* and the *OXTR* genes (respectively, rs3796863 and rs53576).

## Materials and Methods

### Participants

In the present study, psychological and genetic testing was carried out on 298 adolescents aged 12–18 from unbiased urban school samples. The ethnicity of all adolescents included in the study is Russian Caucasoid.

### Procedure

The research was carried out in public education schools after the end of the lessons. Each school was randomly assigned for testing. After receiving informed consent from parents, schoolchildren were notified of the voluntariness and confidentiality of the study. Participants were asked to complete a questionnaire that included demographic data (gender, age, the mother's nationality) and one-sided self-rated SDQ for adolescents aged 11–17. In our sample, twelve adolescents (4%) were 18-years-olds and in the same grade as 17-year-olds; we assume that their psychological characteristics were very similar. Adolescents were asked to provide saliva samples in special containers after filling out the form.

The SDQ consists of 25 statements regarding problematic and socially approved behavior for assessment in adolescents over the prior 6 months. Answers are assessed on a 3-point scale [0 = not true, 1 = somewhat true, and 2 = certainly true; points were assigned in forward or reverse order for each question following the instructions of the authors of the questionnaire (22)] and were grouped according to five scales: emotional symptoms, conduct problems, hyperactivity/inattention, peer problems, and prosocial behavior.

Following the instructions of the authors of the questionnaire (22), the scores for the statements were summed and grouped to calculate the indicator for each scale: emotional symptoms are characterized by statements No 3, 8, 13, 16, and 24; conduct problems are summed from the points of No 5, 7, 12, 18, and 22; hyperactivity/inattention are reflected by statements No 2, 10, 5, 21, and 25, while peer problems are determined from questions No 6, 11, 14, 19, and 23. The total number of points reflects the severity of problems in a particular area for a teenager. Additionally, the scores of the first four scales form another scale named “total difficulties score.” The prosocial behavior score is calculated separately based on the sum of points for statements No 1, 4, 9, 17, and 20. We used the Russian version of the questionnaire for our study, which can be freely downloaded from the developers' site (https://sdqinfo.org/).

Previously, with using factor analysis, some studies have shown that internalizing problems are defined as a combination of SDQ assessed emotional symptoms and peer problems, while externalizing problems are defined as a combination of conduct problems and hyperactivity-inattention (28–30). In our study, we also used this approach.

The study was approved by the Ethics Committee of the Federal Research Center “Krasnoyarsk Science Center of the Siberian Branch of the Russian Academy of Sciences.”

### Genotyping

Saliva samples for genotyping were collected using “Saliva DNA Collection and Preservation Devices” (Norgen Biotek Corp., Thorold, ON Canada). Genomic DNA was extracted from the sample using “DIAtom DNA Prep kits” (IzoGen, Russia). Variants were determined by real-time polymerase chain reaction (RTime-PCR) using “Rotor-Gene 6000” (Corbett Life Science, Australia). Genotyping was carried out using TaqMan allele discrimination technology and commercially available TaqMan probes (DNA-Synthesis, Russia). The PCR reaction system, with a total volume of 25 μL, contained 1 μL of DNA template (about 10 ng), 10 μL of 2.5 × reaction mix for RTime-PCR, 2 μL of 25 mM MgCl2, 8.5 μL of ddH2O (M-428, Syntol, Russia), 2.5 μL of 10 μM primer mix, and 1 μL of each fluorescent probe (DNA-Synthesis, Russia). RTime-PCR conditions were as follows: 95°C for 3 min; 95°C for 20 s, 55°C for 30 s, and 72°C for 20 s (50 cycles). More details related to genotyping are shown in Supplementary Material.

### Statistical Analyses and Power

Statistical analysis was performed using Statistica v.12.0 software (StatSoft Inc., USA). SDQ score data are shown as medians (25–75% quartiles). Differences in categorical data were assessed using the two-tailed Fisher's exact test. The Mann–Whitney U test was used to determine whether there are differences in SDQ scores between groups according to genotypes.

Based on an estimated among Central-North Europoid populations AA+AC/CC genotypes prevalence for CD38 rs3796863 (47/53%), and AA+AG/GG for OXTR rs53576 (63/37%) according to data on http://www.ensembl.org/ and estimated proportion differences ≈ 20% (31), at least 280 participants were needed to have 0.80 power to detect a medium size effect (Cramer's V = 0.3) at α = 0.0083 (with a Bonferroni correction (0.05/6) to adjust for multiple hypothesis testing). Statistical power was tested with the public domain software G^*^Power 3.1.9.2 (32).

## Results

Descriptive statistics for the major study variables, SDQ scales, *CD38* rs3796863, and *OXTR* rs53576 are presented in Table 1. Girls in our sample group showed higher scores in the SDQ scales of emotional symptoms and prosocial behavior compared to boys. Similar gender differences have been described for other populations (26, 33). The frequencies of the *CD38* rs3796863 and *OXTR* rs53576 genotype distribution in the sample group are comparable to their frequencies in Caucasoid populations (according to data on http://www.ensembl.org/) and do not depend on the gender of participants. The allelic distributions for *CD38* rs3796863 and *OXTR* rs53576 are in line with the Hardy-Weinberg equilibrium (please see Supplementary Material).

**Table 1:** Descriptive statistics for major study variables, SDQ scales, *CD38* rs3796863, and *OXTR* rs53576.

**Variables**	**All participants**	**Boys**	**Girls**	***p* (Boys vs. Girls)**
Age 12–14	139	51	88	–
Age 15–18	159	62	97	–
Total	298	113	185	–
**SDQ scales**				
Total difficulties score	12 (8–16)	10 (6–13)	13 (8–13)	<0.001
Conduct problems score	2 (1–3)	2 (1–3)	2 (1–3)	0.286
Emotional symptoms score	3 (1–6)	2 (0–4)	4 (1–6)	<0.001
Hyperactivity score	3 (2–5)	3 (2–5)	3 (2–5)	0.099
Peer problem score	3 (1–4)	2 (1–4)	3 (1–4)	0.429
Prosocial behavior score	7 (6–9)	7 (5–8)	8 (6–9)	<0.001
***CD38*** **rs3796863 genotypes**				
AA	33 (11%)	16 (14%)	17 (9%)	0.179
AC	113 (38%)	39 (35%)	74 (40%)	0.389
CC	152 (51%)	74 (51%)	94 (51%)	1.000
***OXTR*** **rs53576 genotypes**				
AA	48 (16%)	20 (18%)	28 (15%)	0.500
AG	142 (48%)	51 (45%)	91 (49%)	0.502
GG	108 (36%)	42 (37%)	66 (36%)	0.862

*Data for the SDQ are presented as medians (25–75% quartiles) of SDQ scale points. The Mann–Whitney U test was used for SDQ points and two-tailed Fisher's exact test for genotypes*.

As is the case for a large number of similar studies that evaluated genotypic differences for the rs3796863 variant (34–38), we used the dominant inheritance model to ensure a sufficient number of participants in each group to be analyzed, where the minor homozygotes and heterozygotes for *CD38* rs3796863 (AA and AC) were combined and compared with the homozygotes for the major allele (CC). The same analysis strategy was used to assess the *OXTR* rs53576 genotypes: minor homozygotes and heterozygotes (AA and AG) were combined and compared with homozygotes for the major allele (GG), which was also used in several studies of Caucasoid populations, in which the G allele predominates (in contrast to Asian populations, in which the A allele is more common) (39–42).

Table 2 presents the *CD38* rs3796863 and *OXTR* rs53576 genotypes distributions according to SDQ results. There was no evidence of genotype differences in the Prosocial behavior score and Internalizing problems. The carriage of high-OT-producing *CD38* rs3796863 genotypes (AA + AC) exhibits only a weak tendency toward the lower level of Externalizing problems (*p* = 0.058) that substantially exceeded the Bonferroni adjustment of *p* < 0.0083.

**Table 2:** The *CD38* rs3796863 and *OXTR* rs53576 genotypes distributions according to SDQ results.

**Genotypes**	**Prosocial behavior score**			**Internalizing problems**			**Externalizing problems**		
	**>5** **(*n* = 237)**	**≤5** **(*n* = 61)**	***p***	**No** **(*n* = 239)**	**Yes** **(*n* = 59)**	***p***	**No** **(*n* = 181)**	**Yes** **(*n* = 117)**	***p***
*CD38* rs3796863AA+AC	116 (49%)	30 (49%)	1.000	118 (49%)	28 (48%)	0.885	97 (53%)	49 (42%)	0.058
*CD38* rs3796863CC	121 (51%)	31 (51%)		121 (51%)	31 (52%)		84 (47%)	68 (58%)	
*OXTR*rs53576AA+AG	153 (65%)	37 (61%)	0.654	150 (63%)	40 (68%)	0.546	117 (65%)	73 (62%)	0.712
*OXTR*rs53576GG	84 (35%)	24 (39%)		89 (37%)	19 (32%)		64 (35%)	44 (38%)	

*Data for the SDQ results are presented as n (%). Two-tailed Fisher's exact test was used for genotypes*.

It is known that oxytocin production decreases in adolescents in comparison with pre-pubertal children, and there are sex differences in OT production: levels are higher in adolescent girls and women (43, 44). Moreover, the median values of individual SDQ scales for boys significantly differ from that of the girls in our sample group (Table 1). To take into consideration such age and gender differences, we carried out the *post-hoc* discrete analysis of the *CD38* rs3796863 and *OXTR* rs53576 genotype influences on the indicators of the SDQ questionnaire scales in two age groups (12–14 and 15–18 years old) separately for boys and girls. The median values of SDQ scales according to the different *CD38* rs3796863 and *OXTR* rs53576 genotypes are, respectively, presented in Tables 3, 4.

**Table 3:** Values of the SDQ scales for the *CD38* rs3796863 genotypes in different sex and age groups of adolescents.

**SDQ scales**	***CD38*** **rs3796863 genotypes**											
	**Age 12–14**						**Age 15–18**					
	**Boys (** ***n*** **=** **51)**			**Girls (** ***n*** **=** **88)**			**Boys (** ***n*** **=** **62)**			**Girls (** ***n*** **=** **97)**		
	**AA+AC** **(*n* = 21)**	**CC** **(*n* = 30)**	***p***	**AA+AC** **(*n* = 40)**	**CC** **(*n* = 48)**	***p***	**AA+AC** **(*n* = 34)**	**CC** **(*n* = 28)**	***p***	**AA+AC** **(*n* = 46)**	**CC** **(*n* = 51)**	***p***
Total difficulties score	11 (8–13)	10 (9–16)	0.840	16 (13–19.5)	14 (9–17)	0.053	9 (5–12)	9 (4.5–14)	0.843	11 (7–13)	11.5 (8–17)	0.585
Conduct problems score	3 (2–3)	2 (1–3)	0.066	3 (2–4)	3 (1–5)	0.996	2 (2–3)	2 (1–2)	0.078	2 (1–3)	2 (1–3)	0.973
Emotional symptoms score	2 (0–3)	2 (0–4)	0.401	6 (4–7)	3 (2–7)	0.022	2 (0–3)	1.5 (0–3)	0.628	3 (2–5)	4 (1–6)	0.847
Hyperactivity score	3 (2–5)	3.5 (2–5)	0.816	4.5 (3–6)	3.5 (2–6)	0.102	3 (1–4)	3 (1–6)	0.453	3 (1–5)	3 (1–5)	0.895
Peer problem score	3 (2–4)	3 (2–4)	0.976	3.5 (2–5)	3 (1.5–4)	0.266	2 (1–3)	2 (1–4)	0.908	2 (1–4)	3 (2–4)	0.101
Prosocial behavior score	6 (5–8)	7 (5–8)	0.353	8 (6.5–9)	7 (6–8)	0.075	6.5 (4–8)	7 (5.5–8)	0.493	8 (7–9)	8 (7–9)	0.716

*Data are presented as medians (25–75% quartiles) of SDQ scale points. The Mann–Whitney U test was used*.

**Table 4:** Values of the SDQ scales for the *OXTR* rs53576 genotypes in different sex and age groups of adolescents.

**SDQ scales**	***OXTR rs53576*** **genotypes**											
	**Age 12–14**						**Age 15–18**					
	**Boys (** ***n*** **=** **51)**			**Girls (** ***n*** **=** **88)**			**Boys (** ***n*** **=** **62)**			**Girls (** ***n*** **=** **97)**		
	**AA+AG** **(*n* = 30)**	**GG** **(*n* = 21)**	***p***	**AA+AG** **(*n* = 51)**	**GG** **(*n* = 37)**	***p***	**AA+AG** **(*n* = 41)**	**GG** **(*n* = 21)**	***p***	**AA+AG** **(*n* = 68)**	**GG** **(*n* = 29)**	***p***
Total difficulties score	11 (8–16)	10 (9–12)	0.323	15 (12–19)	15 (9–18)	0.573	8 (4–12)	11 (8–13)	0.050	11 (7.5–14)	11 (7–16)	0.850
Conduct problems score	2 (1–3)	2 (2–3)	0.944	3 (2–4)	3 (2–4)	0.403	2 (1–3)	2 (1–2)	0.286	2 (1–3)	2 (1–3)	0.661
Emotional symptoms score	1,5 (0–4)	2 (2–4)	0.822	5 (2–7)	5 (2–7)	0.752	1 (0–2)	3 (2–4)	0.004	3.5 (2–5)	3 (1–6)	0.680
Hyperactivity score	4 (2–5)	3 (2–5)	0.283	4 (2–5)	4 (3–6)	0.310	1 (0–2)	3 (2–4)	0.103	3 (1–5)	3 (1–5)	0.824
Peer problem score	3 (2–5)	3 (2–3)	0.325	3 (2–5)	3 (1–4)	0.156	2 (1–3)	3 (1–4)	0.586	3 (1–4)	3 (2–4)	0.677
Prosocial behavior score	7 (5–8)	6 (6–8)	0.810	7 (6–8)	7 (6–9)	0.983	7 (4–8)	7 (5–8)	0.314	8 (7–9)	8 (7–9)	0.310

*Data are presented as medians (25–75% quartiles) of SDQ scale points. The Mann–Whitney U test was used*.

The tendency toward an association between *CD38* rs3796863 genotypes and SDQ scores was found in girls aged 12–14 years (Table 3). In this group of adolescents, carriage of the high-OT-producing genotypes (AA + AC) was associated with high values of the Emotional symptoms score (*p* = 0.022, Bonferroni adjusted *p* = 0.176). SDQ score analysis based on *OXTR* rs53576 genotypes showed a statistically significant association only in the subgroup of older (15–18 years old) adolescent boys (Table 4). Homozygosity for the G allele, which is presumably associated with high activity of the oxytocin receptor, was associated with the presence of emotional problems (*p* = 0.004, Bonferroni adjusted *p* = 0.032).

## Discussion

Variationsat the *CD38* rs3796863 and *OXTR* rs53576 loci were not associated with psychosocial characteristics of adolescents assessed with SDQ in our complete sample. Our results are similar to the recent findings from Conner et al. study that found no correlation between *OXTR* rs53576 genetic polymorphism and emotional traits, including depressive symptoms, psychological well-being, optimism, and self-esteem in young adults (45). Another study of 10,760 participants from 2017 found no role of the variants within the *OXTR* gene in loneliness, a trait correlated with neuroticism and depressive symptoms; however, this study found that these traits do show heritability, but they are highly polygenic (46). McInnis et al. investigated the same polymorphism for the *CD38* and *OXTR* genes (rs3796863 and rs53576) found no relation of *OXTR* and unsupportive social interactions and affective states in undergraduate students (*n* = 476), but A carriers of the *CD38* polymorphism exhibited greater perceived peer unsupportive interactions compared to CC carriers (37). Conversely, Huetter et al. established a lack of association of the *CD38* rs3796863 polymorphisms and a significant association of the OXTR rs53576 variants linked to empathic behavior in 421 healthy adults (47). No link the *CD38* rs3796863 variants with emotion perception in 120 patients with anorexia nervosa were found (31). Lastly, in the Young Finns Study, Dobewall et al. did not find an effect of oxytocin pathway genes (including *CD38* rs3796863) on the initial levels of dispositional compassion for others (48).

In contrast, as mentioned above, many studies have shown significant association of the *CD38* rs3796863 and *OXTR* rs53576 genes polymorphisms with psychosocial and emotional status in adolescents and young adults (6–8, 18–21). Given the conflicting research results, we suppose that replication studies are highly needed with considering gene-environment interaction models, age, and gender characteristics.

The *post-hoc* results of our study show the possible presence of age–sex features of the influence of OT production (*CD38*) and OT reception (*OXTR*) genes on the psychosocial characteristics of adolescents. This is not surprising, as there are two parallel processes in adolescence: the age-dependent decrease in OT production and the emergence of sex differences in its production (43). In addition to these, such differentiation can be influenced by a complex and insufficiently studied interaction between OT and the entire spectrum of sex hormones, exhibiting highly dynamic changes in adolescence (49).

We presume that a relatively large production of OT, mediated by the carriage of the A allele of the rs3796863 variant region of the *CD38* gene, may be associated with disturbances in the emotional sphere in young adolescent girls due to a higher level of social sensitivity, which corresponds with the results of other studies. The hypothesis of CD38-mediated social sensitivity as a general mechanism of oxytocin moderation of an increased psycho-emotional response to positive and negative social stimuli was originally put forward by Bartz and McInnes (50, 51). This hypothesis was later confirmed. For example, in the examination of 400 adolescents, Tabak et al. showed that carriers of the A allele rs3796863 of the *CD38* gene were more sensitive to chronic interpersonal stress than CC homozygotes (35). Mediated by genetic variations in *CD38*, high levels of social sensitivity as a factor of emotional problems were demonstrated in a study by McQuaid et al. (52). The authors conducted genetic testing of 19-year-old students and found that AA homozygotes of rs3796863 experienced heightened feelings of alienation from parents and peers, symptoms of depression, and an increased level of suicidal ideation. Later, the same authors showed that carriers of the A allele of rs3796863 were more sensitive to unsupportive social interactions with their peers (relationships that bring pain, suffering, sadness, isolation, rejection, and troubles) (37). Lebowitz et al. found that the influence of negative relationships with peers more often led to suicidal ideations in adolescents with high OT levels in saliva, which also supports the hypothesis of OT-mediated excessive social sensitivity (53).

According to our *post-hoc* data, the mechanisms of OT reception, mediated by the carriage of the G allele of the *OXTR* rs53576 gene, may be more vital for older adolescent boys in the regulation of emotions and behavior, as this age–sex group experiences the conditions of relatively low OT production in comparison with girls and younger boys. The provocative role of G-allele carriage in the formation of psycho-emotional problems in adolescents has been described in several studies. For example, Smearman et al. revealed a greater level of behavioral problems under the influence of stress factors in adolescents with the G allele of rs53576 (54). McQuaid et al. described the association of G allele of rs53576 carriage with depressive symptoms in students who underwent a traumatic situation in childhood (55). The authors considered these results to be paradoxical since a large number of studies have simultaneously shown the association of the G allele of rs53576 with extremely socially useful qualities, such as empathy, optimism, and trust (19, 21, 39, 56, 57). As a possible explanation for their findings, the authors proposed the hypothesis of excessive social sensitivity in individuals with high production and reception of OT, which is described above. Similar results were later obtained by Hostinar et al.: in adolescents who experienced childhood maltreatment, a high level of anxiety and depression symptoms were more often found in those with homozygosity for GG of rs53576 (58).

Some studies confirm the existence of sex differences in the regulation of OT reception in adolescent populations. Thus, Andreou et al. conducted genetic testing of 1,591 Swedish adolescents and revealed an association between the G allele of the rs53576 region of the *OXTR* gene and antisocial behavior in maltreatment cases, but this was still only applicable to girls, not boys (9). The authors concluded that it is mandatory to consider the gender factor in studies on the role of the oxytocinergic system in adolescents. In a prospective study of 593 15-year-old Estonian adolescents, it was shown that in boys (but not girls), homozygosity for the allelic variant A of rs53576 was associated with more frequent alcohol consumption and the development of addiction to alcohol by the age of 25 (59). It was found that in a Chinese population of adolescents homozygous for the major allele in Asian populations, which is the A rs53576 *OXTR* allele, there is a greater level of hostility and aggressiveness when the individual is exposed to stress factors (7). The effect was much more typical for boys than for girls, which is consistent with our data.

It is known that oxytocin receptors are concentrated mainly in the amygdala, and the size of the amygdala in men is larger than that in women, on average. It is negatively correlated with prosocial behavior. In this regard, the data of Tost et al. found that the right amygdala was smaller in homozygous carriers of the G allele of the *OXTR* rs53576 gene (typical only for men, not women), are extremely interesting (60). There are well-known data on sex differences in psychological reactions to the administration of exogenous OT, which were obtained both in experiments with animals and in controlled studies in humans (49, 61). Lucas-Thompson et al. investigated the psychological response to the September 11 terrorist attack and found that the *OXTR* rs53576 polymorphism moderated the stress response only in men, but not in women, and homozygosity for GG of rs53576 was associated with poorer stress tolerance (41). In men, but not in women, GG homozygosity of rs53576 was associated with an increased sympathetic response of the cardiorespiratory system to stress compared to carriers of the A allele (62). Finally, Nishina et al. found a higher confidence level in carriers of the GG genotype of rs53576 variant in Japanese men but not women (42). The results of the studies mentioned above correspond well enough with our data on the greater effect of the *OXTR* rs53576 gene variant on the psychosocial characteristics of boys, but not girls, and this phenomenon manifested only in older adolescents in the process of growing up. It may be assumed that the differences in the structure and function of the amygdala are fully formed only after reaching older adolescence.

Some limitations characterize the present study. Over the past few years, genome-wide association studies with hundreds of thousands of participants have shown that individual candidate genes do not explain sufficient variance in complex human psychological and behavioral traits. For example, a study from 2019 with very large samples ranging from 62,138 to 443,264 participants showed no main effect or G x E interactions of single candidate genes on depressive symptoms, which are closely related to psychosocial problems, the construct in the current study (63). Thus, findings like in our *post-hoc* analysis are likely to be false positives due to small subgroups size. For reducing the chance of reporting false-positive/negative associations for our main group, we conducted our study in an unbiased school sample and previously calculated the required sample size. In many other similar studies with the null findings for rs3796863 and rs53576, the number of participants varies from 120 to 480 (31, 37, 45, 48, 64, 65). In the present study, we enrolled 298 adolescents, which were sufficient for statistical power 0.8 for our main group and according to our design. The SDQ allows assessing the psychosocial state of adolescents exclusively over the prior 6 months and does not reflect life-time problems; it might not be suitable for genetic studies. With regards to this issue is more reasonable to conduct a longitudinal study (48) which also significantly increases the statistical power.

In studies with a similar design, we extremely recommend replication in larger samples with greater power to detect small effects, especially in subgroups. We also suppose that the psychophysiological role of OT should be assessed in the context of a social environment, ethnocultural factors, age, and gender characteristics (e.g., stratification by age and sex should be mandatory in this type of study among adolescents).

## Data Availability Statement

The original contributions presented in the study are included in the article/Supplementary Material, further inquiries can be directed to the corresponding author/s.

## Ethics Statement

The studies involving human participants were reviewed and approved by the Ethics Committee of the Federal Research Center Krasnoyarsk Science Center of the Siberian Branch of the Russian Academy of Sciences. Written informed consent to participate in this study was provided by the participants' legal guardian/next of kin.

## Author Contributions

ST, EK, and NS: conceptualization. SZ, MSm, LE, OZ, MSh, NG, and LL: investigation. MSh: data curation. ST and SZ: writing—original draft preparation and writing—review and editing. ST: project administration and funding acquisition. All authors have read and agreed to the published version of the manuscript.

## Conflict of Interest

The authors declare that the research was conducted in the absence of any commercial or financial relationships that could be construed as a potential conflict of interest.

## Publisher's Note

All claims expressed in this article are solely those of the authors and do not necessarily represent those of their affiliated organizations, or those of the publisher, the editors and the reviewers. Any product that may be evaluated in this article, or claim that may be made by its manufacturer, is not guaranteed or endorsed by the publisher.

## Abbreviations

OT: Oxytocin
CD38: Cluster of differentiation 38
OXTR: Oxytocin receptor
SDQ: The Strengths and Difficulties Questionnaire
DNA: Deoxyribonucleic acid
RT-PCR: Real-time polymerase chain reaction
HWE: Hardy–Weinberg equilibrium.

## References

[1] Torres N Martins D Santos AJ Prata D Verissimo M. How do hypothalamic nonapeptides shape youth's sociality? A systematic review on oxytocin, vasopressin and human socio-emotional development. Neurosci Biobehav Rev. (2018) 90:309–31. 10.1016/j.neubiorev.2018.05.004 29738796

[2] Kavaliers M Matta R Choleris E. Mate-choice copying, social information processing, and the roles of oxytocin. Neurosci Biobehav Rev. (2017) 72:232–42. 10.1016/j.neubiorev.2016.12.003 27923732

[3] Plessow F Eddy KT Lawson EA. The neuropeptide hormone oxytocin in eating disorders. Curr Psychiatry Rep. (2018) 20:91. 10.1007/s11920-018-0957-0 30178087 PMC6903388

[4] Xu S Qin B Shi A Zhao J Guo X Dong L. Oxytocin inhibited stress induced visceral hypersensitivity, enteric glial cells activation, and release of proinflammatory cytokines in maternal separated rats. Eur J Pharmacol. (2018) 818:578–84. 10.1016/j.ejphar.2017.11.018 29162434

[5] Rash JA Aguirre-Camacho A Campbell TS. Oxytocin and pain: a systematic review and synthesis of findings. Clin J Pain. (2014) 30:453–62. 10.1097/AJP.0b013e31829f57df 23887343

[6] Feldman R Monakhov M Pratt M Ebstein RP. Oxytocin pathway genes: evolutionary ancient system impacting on human affiliation, sociality, and psychopathology. Biol Psychiatry. (2016) 79:174–84. 10.1016/j.biopsych.2015.08.008 26392129

[7] Shao D Zhang HH Long ZT Li J Bai HY Li JJ. Effect of the interaction between oxytocin receptor gene polymorphism (rs53576) and stressful life events on aggression in Chinese Han adolescents. Psychoneuroendocrinology. (2018) 96:35–41. 10.1016/j.psyneuen.2018.06.002 29890447

[8] Parris MS Grunebaum MF Galfalvy HC Andronikashvili A Burke AK Yin H. Attempted suicide and oxytocin-related gene polymorphisms. J Affect Disord. (2018) 238:62–8. 10.1016/j.jad.2018.05.022 29860184

[9] Andreou D Comasco E Aslund C Nilsson KW Hodgins S. Maltreatment, the oxytocin receptor gene, and conduct problems among male and female teenagers. Front Hum Neurosci. (2018) 12:112. 10.3389/fnhum.2018.00112 29623035 PMC5874495

[10] Tereshchenko SY Smolnikova M. Oxitocin is a hormone of trust and emotional attachment: the influence on behavior of children and adolescents. Zhurnal nevrologii i psikhiatrii imeni SS Korsakova. (2019) 119:148–53. 10.17116/jnevro2019119121148 31994529

[11] Ayaz AB Karkucak M Ayaz M Gokce S Kayan E Guler EE. Oxytocin system social function impacts in children with attention-deficit/hyperactivity disorder. Am J Med Genet B Neuropsychiatr Genet. (2015) 168:609–16. 10.1002/ajmg.b.32343 26174935

[12] Cataldo I Azhari A Lepri B Esposito G. Oxytocin receptors (OXTR) and early parental care: an interaction that modulates psychiatric disorders. Res Dev Disabil. (2018) 82:27–38. 10.1016/j.ridd.2017.10.007 29033100

[13] Higashida H Hashii M Tanaka Y Matsukawa S Higuchi Y Gabata R. CD38, CD157, and RAGE as molecular determinants for social behavior. Cells. (2019) 9:62. 10.3390/cells9010062 31881755 PMC7016687

[14] Jin D Liu H.-X. Hirai H Torashima T Nagai T. CD38 is critical for social behaviour by regulating oxytocin secretion. Nature. (2007) 446:41–45. 10.1038/nature05526 17287729

[15] Liu H.-X. Lopatina O Higashida C Tsuji T Kato I. Locomotor activity, ultrasonic vocalization and oxytocin levels in infant CD38 knockout mice. Neurosci Lett. (2008) 448:67–70. 10.1016/j.neulet.2008.09.084 18926879

[16] Malavasi F Deaglio S Funaro A Ferrero E Horenstein AL Ortolan E. Evolution and function of the ADP ribosyl cyclase/CD38 gene family in physiology and pathology. Physiol Rev. (2008) 88:841–86. 10.1152/physrev.00035.2007 18626062

[17] Feldman R Zagoory-Sharon O Weisman O Schneiderman I Gordon I Maoz R. Sensitive parenting is associated with plasma oxytocin and polymorphisms in the OXTR and CD38 genes. Biol Psychiatry. (2012) 72:175–81. 10.1016/j.biopsych.2011.12.025 22336563

[18] Bell AF Carter CS Steer CD Golding J Davis JM Steffen AD. Interaction between oxytocin receptor DNA methylation and genotype is associated with risk of postpartum depression in women without depression in pregnancy. Front Genet. (2015) 6:243. 10.3389/fgene.2015.00243 26257770 PMC4508577

[19] Li J Zhao Y Li R Broster LS Zhou C Yang S. Association of oxytocin receptor gene (OXTR) rs53576 polymorphism with sociality: a meta-analysis. PLoS ONE. (2015) 10:e0131820. 10.1371/journal.pone.0131820 26121678 PMC4488068

[20] Reiner I Van Ijzendoorn MH Bakermans-Kranenburg MJ Bleich S Beutel M Frieling H. Methylation of the oxytocin receptor gene in clinically depressed patients compared to controls: the role of OXTR rs53576 genotype. J Psychiatr Res. (2015) 65:9–15. 10.1016/j.jpsychires.2015.03.012 25890851

[21] Gong P Fan H Liu J Yang X Zhang K Zhou X. Revisiting the impact of OXTR rs53576 on empathy: a population-based study and a meta-analysis. Psychoneuroendocrinology. (2017) 80:131–6. 10.1016/j.psyneuen.2017.03.005 28343138

[22] Goodman R Meltzer H Bailey V. The strengths and difficulties questionnaire: a pilot study on the validity of the self-report version. Eur Child Adolesc Psychiatry. (1998) 7:125–30. 10.1007/s007870050057 9826298

[23] Silva TB Osório FL Loureiro SR. SDQ: discriminative validity and diagnostic potential. Front Psychol. (2015) 6:811. 10.3389/fpsyg.2015.00811 26113840 PMC4462033

[24] Hall CL Guo B Valentine AZ Groom MJ Daley D Sayal K. The validity of the strengths and difficulties questionnaire (SDQ) for children with ADHD symptoms. PLoS ONE. (2019) 14:e0218518. 10.1371/journal.pone.0218518 31216327 PMC6583960

[25] Vugteveen J de Bildt A Theunissen M Reijneveld M Timmerman M. Validity aspects of the strengths and difficulties questionnaire (SDQ) adolescent self-report and parent-report versions among Dutch adolescents. Assessment. (2021) 28:601–16. 10.1177/1073191119858416 31257902 PMC7883005

[26] Ruchkin V Koposov R Schwab-Stone M. The strength and difficulties questionnaire: scale validation with Russian adolescents. J Clin Psychol. (2007) 63:861–9. 10.1002/jclp.20401 17674399

[27] Slobodskaya HR Akhmetova OA Ryabichenko TI. Siberian child and adolescent mental health: prevalence estimates and psychosocial factors. Alaska Med. (2007) 49:261–6. 17929646

[28] Koskelainen M Sourander A Vauras M. Self-reported strengths and difficulties in a community sample of Finnish adolescents. Eur Child Adolesc Psychiatry. (2001) 10:180–5. 10.1007/s007870170024 11596818

[29] Dickey WC Blumberg SJ. Revisiting the factor structure of the strengths and difficulties questionnaire: United States, 2001. J Am Acad Child Adolesc Psychiatry. (2004) 43:1159–67. 10.1097/01.chi.0000132808.36708.a9 15322420

[30] Choi D Tsuchiya KJ Takei N. Interaction effect of oxytocin receptor (OXTR) rs53576 genotype and maternal postpartum depression on child behavioural problems. Sci Rep. (2019) 9:7685. 10.1038/s41598-019-44175-6 31118457 PMC6531431

[31] Kucharska K Kot E Biernacka K Zimowski J Rogoza R Rybakowski F. Interaction between polymorphisms of the oxytocinergic system genes and emotion perception in inpatients with anorexia nervosa. Eur Eating Disord Rev. (2019) 27:481–94. 10.1002/erv.2698 31385420

[32] Faul F Erdfelder E Buchner A Lang AG. Statistical power analyses using G^*^Power 3.1: tests for correlation and regression analyses. Behav Res Methods. (2009) 41:1149–60. 10.3758/BRM.41.4.1149 19897823

[33] Kaiser S Kyrrestad H Fossum S. Cyberbullying status and mental health in Norwegian adolescents. Scand J Psychol. (2020) 61:707–13. 10.1111/sjop.12656 32592170

[34] Sauer C Montag C Reuter M Kirsch P. Imaging oxytocin × dopamine interactions: an epistasis effect of CD38 and COMT gene variants influences the impact of oxytocin on amygdala activation to social stimuli. Front Neurosci. (2013) 7:45. 10.3389/fnins.2013.00045 23554586 PMC3612689

[35] Tabak BA Vrshek-Schallhorn S Zinbarg RE Prenoveau JM Mineka S Redei EE. Interaction of CD38 variant and chronic interpersonal stress prospectively predicts social anxiety and depression symptoms over 6 years. Clin Psychol Sci. (2016) 4:17–27. 10.1177/2167702615577470 26958455 PMC4779340

[36] Liu J Gong P Li H Zhou X. A field study of the association between CD38 gene and altruistic behavior: empathic response as a mediator. Psychoneuroendocrinology. (2017) 85:165–71. 10.1016/j.psyneuen.2017.08.010 28865941

[37] McInnis OA McQuaid RJ Matheson K Anisman H. Unsupportive social interactions and affective states: examining associations of two oxytocin-related polymorphisms. Stress. (2017) 20:122–9. 10.1080/10253890.2017.1286326 28235397

[38] Tabak BA Young KS Torre JB Way BM Burklund LJ Eisenberger NI. Preliminary evidence that CD38 moderates the association of neuroticism on amygdala-subgenual cingulate connectivity. Front Neurosci. (2020) 14:11. 10.3389/fnins.2020.00011 32116489 PMC7033443

[39] Rodrigues SM Saslow LR Garcia N John OP Keltner D. Oxytocin receptor genetic variation relates to empathy and stress reactivity in humans. Proc Natl Acad Sci. (2009) 106:21437–41. 10.1073/pnas.0909579106 19934046 PMC2795557

[40] Tops M Van Ijzendoorn MH Riem MME Boksem MAS Bakermans-Kranenburg MJ. Oxytocin receptor gene associated with the efficiency of social auditory processing. Front Psychiatry. (2011) 2:60. 10.3389/fpsyt.2011.00060 22069391 PMC3208383

[41] Lucas-Thompson RG Holman EA. Environmental stress, oxytocin receptor gene (OXTR) polymorphism, and mental health following collective stress. Horm Behav. (2013) 63:615–24. 10.1016/j.yhbeh.2013.02.015 23470776

[42] Nishina K Takagishi H Inoue-Murayama M Takahashi H Yamagishi T. Polymorphism of the oxytocin receptor gene modulates behavioral and attitudinal trust among men but not women. PLoS ONE. (2015) 10:e0137089. 10.1371/journal.pone.0137089 26444016 PMC4621758

[43] Doom JR Doyle CM Gunnar MR. Social stress buffering by friends in childhood and adolescence: effects on HPA and oxytocin activity. Soc Neurosci. (2017) 12:8–21. 10.1080/17470919.2016.1149095 26899419 PMC5538015

[44] Marazziti D Baroni S Mucci F Piccinni A Moroni I Giannaccini G. Sex-related differences in plasma oxytocin levels in humans. Clin Pract Epidemiol Ment Health. (2019) 15:58–63. 10.2174/1745017901915010058 31015856 PMC6446474

[45] Conner TS McFarlane KG Choukri M Riordan BC Flett JAM Phipps-Green AJ. The oxytocin receptor gene (OXTR) variant rs53576 Is not related to emotional traits or states in young adults. Front Psychol. (2018) 9:2548. 10.3389/fpsyg.2018.02548 30618969 PMC6297827

[46] Gao J Davis LK Hart AB Sanchez-Roige S Han L Cacioppo JT. Genome-wide association study of loneliness demonstrates a role for common variation. Neuropsychopharmacology. (2017) 42:811–21. 10.1038/npp.2016.197 27629369 PMC5312064

[47] Huetter FK Bachmann HS Reinders A Siffert D Stelmach P Knop D. Association of a common oxytocin receptor gene polymorphism with self-reported ‘empathic concern' in a large population of healthy volunteers. PLoS ONE. (2016) 11:e0160059. 10.1371/journal.pone.0160059 27467763 PMC4965009

[48] Dobewall H Saarinen A Lyytikäinen L.-P. Keltikangas-Järvinen L Lehtimäki T. Functional polymorphisms in oxytocin and dopamine pathway genes and the development of dispositional compassion over time: the young finns study. Front Psychol. (2021) 12:576346. 10.3389/fpsyg.2021.576346 33897514 PMC8060576

[49] Macdonald KS. Sex, receptors, and attachment: a review of individual factors influencing response to oxytocin. Front Neurosci. (2013) 6:194. 10.3389/fnins.2012.00194 23335876 PMC3541513

[50] Bartz JA McInnes LA. CD38 regulates oxytocin secretion and complex social behavior. BioEssays. (2007) 29:837–41. 10.1002/bies.20623 17688286

[51] Bartz JA Zaki J Bolger N Ochsner KN. Social effects of oxytocin in humans: context and person matter. Trends Cogn Sci. (2011) 15:301–9. 10.1016/j.tics.2011.05.002 21696997

[52] McQuaid RJ McInnis OA Matheson K Anisman H. Oxytocin and social sensitivity: gene polymorphisms in relation to depressive symptoms and suicidal ideation. Front Hum Neurosci. (2016) 10:358. 10.3389/fnhum.2016.00358 27486392 PMC4949220

[53] Lebowitz ER Blumberg HP Silverman WK. Negative peer social interactions and oxytocin levels linked to suicidal ideation in anxious youth. J Affect Disord. (2019) 245:806–11. 10.1016/j.jad.2018.11.070 30699863 PMC6361537

[54] Smearman EL Winiarski DA Brennan PA Najman J Johnson KC. Social stress and the oxytocin receptor gene interact to predict antisocial behavior in an at-risk cohort. Dev Psychopathol. (2015) 27:309–18. 10.1017/S0954579414000649 25003328 PMC5506377

[55] McQuaid RJ McInnis OA Stead JD Matheson K Anisman H. A paradoxical association of an oxytocin receptor gene polymorphism: early-life adversity and vulnerability to depression. Front Neurosci. (2013) 7:128. 10.3389/fnins.2013.00128 23898235 PMC3721019

[56] Saphire-Bernstein S Way BM Kim HS Sherman DK Taylor SE. Oxytocin receptor gene (OXTR) is related to psychological resources. Proc Natl Acad Sci. (2011) 108:15118–22. 10.1073/pnas.1113137108 21896752 PMC3174632

[57] Krueger F Parasuraman R Iyengar V Thornburg M Weel J Lin M. Oxytocin receptor genetic variation promotes human trust behavior. Front Hum Neurosci. (2012) 6:4. 10.3389/fnhum.2012.00004 22347177 PMC3270329

[58] Hostinar CE Cicchetti D Rogosch FA. Oxytocin receptor gene polymorphism, perceived social support, and psychological symptoms in maltreated adolescents. Dev Psychopathol. (2014) 26:465–77. 10.1017/S0954579414000066 24621832 PMC4141414

[59] Vaht M Kurrikoff T Laas K Veidebaum T Harro J. Oxytocin receptor gene variation rs53576 and alcohol abuse in a longitudinal population representative study. Psychoneuroendocrinology. (2016) 74:333–41. 10.1016/j.psyneuen.2016.09.018 27716573

[60] Tost H Kolachana B Hakimi S Lemaitre H Verchinski BA Mattay VS. A common allele in the oxytocin receptor gene (OXTR) impacts prosocial temperament and human hypothalamic-limbic structure and function. Proc Natl Acad Sci. (2010) 107:13936–41. 10.1073/pnas.1003296107 20647384 PMC2922278

[61] Van Anders SM Goldey KL Kuo PX. The steroid/peptide theory of social bonds: integrating testosterone and peptide responses for classifying social behavioral contexts. Psychoneuroendocrinology. (2011) 36:1265–75. 10.1016/j.psyneuen.2011.06.001 21724336

[62] Norman GJ Hawkley L Luhmann M Ball AB Cole SW Berntson GG. Variation in the oxytocin receptor gene influences neurocardiac reactivity to social stress and HPA function: a population based study. Horm Behav. (2012) 61:134–9. 10.1016/j.yhbeh.2011.11.006 22146101

[63] Border R Johnson EC Evans LM Smolen A Berley N Sullivan PF. No support for historical candidate gene or candidate gene-by-interaction hypotheses for major depression across multiple large samples. Am J Psychiatry. (2019) 176:376–87. 10.1176/appi.ajp.2018.18070881 30845820 PMC6548317

[64] Davis C Patte K Zai C Kennedy JL. Polymorphisms of the oxytocin receptor gene and overeating: the intermediary role of endophenotypic risk factors. Nutr Diabetes. (2017) 7:e279. 10.1038/nutd.2017.24 28530679 PMC5518806

[65] Huetter FK Moehlendick B Knop D Siffert W. Lack of association of common polymorphisms linked to empathic behavior with self-reported trait empathy in healthy volunteers. Horm Behav. (2020) 126:104841. 10.1016/j.yhbeh.2020.104841 32828797

