# Supplementary material for: Oxytocin Pathway Gene (CD38, OXTR) Variants Are Not Related to Psychosocial Characteristics Defined by Strengths and Difficulties Questionnaire in Adolescents: A Field School-Based Study
Source: Front Psychiatry. 2021 Aug 9;12:714093. doi: 10.3389/fpsyt.2021.714093 (PMC8380924; doi:10.3389/fpsyt.2021.714093)
Supplement: Supplementary file 1 [file Table_1.DOCX]

Supplementary Material

**Supplementary Table S1.** Primer and fluorescent probe sequences.

| Variant | RTime-PCR primers | RTime-PCR probes | Amplicon length (bp) | HWE *p*-value |
| --- | --- | --- | --- | --- |
| *CD38* rs3796863 | Fwd: 5’-CATGTCGGGAGGGGAGCTA-3’  Rev: 5’-GCCTTGGTTGCTGCTCCTG-3’ | FAM-TGACCA**G**CAGGTG-BHQ1  VIC-TTGACCA**T**CAGGTG-BHQ1 | 67 | 0.092 |
| *OXTR* rs53576 | Fwd: 5’-GCATTCATGGAAAGGAAAGGT-3’  Rev: 5’-CCCATCTGTAGAATGAGCTTCC-3’ | FAM-CCCGAGG**A**TCCTCAG -BHQ1  VIC-CCCGAGG**G**TCCTCA-BHQ1 | 94 | 0.908 |
